# Clinical Implementation of Sustainable Functional Foods and Nutraceuticals in Metabolic Health: A Feasibility Study

**DOI:** 10.3390/nu17243858

**Published:** 2025-12-10

**Authors:** Francesca Scionti, Samantha Maurotti, Elisa Mazza, Angela Mirarchi, Raffaella Russo, Paola Doria, Rosario Mare, Giuseppe Marafioti, Yvelise Ferro, Tiziana Montalcini, Arturo Pujia

**Affiliations:** 1 Department of Medical and Surgical Sciences, University Magna Graecia, 88100 Catanzaro, Italy; scionti@unicz.it (F.S.); a.mirarchi@unicz.it (A.M.); raffaella.russo.md@gmail.com (R.R.); mare@unicz.it (R.M.); giuseppe.marafioti1@studenti.unicz.it (G.M.); pujia@unicz.it (A.P.); 2 Department of Clinical and Experimental Medicine, University Magna Graecia, 88100 Catanzaro, Italy; smaurotti@unicz.it (S.M.); paola.doria@unicz.it (P.D.); tmontalcini@unicz.it (T.M.); 3 Research Center for the Prevention and Treatment of Metabolic Diseases, University Magna Graecia, 88100 Catanzaro, Italy

**Keywords:** dietary intervention, functional foods, nutraceuticals

## Abstract

**Background:** Diet quality significantly influences metabolic health, obesity, and non-communicable disease risk. Functional foods and nutraceuticals, rich in bioactive compounds, may enhance health outcomes beyond basic nutrition, particularly when combined with Mediterranean-style dietary patterns. **Objectives:** This feasibility study evaluated the integration of functional foods and nutraceuticals into a Mediterranean-based dietary intervention in adults with metabolic risk factors, focusing on feasibility, tolerability, and preliminary clinical effects. **Methods:** Functional food prototypes, including Calabrian tomato, pomegranate, bergamot, blueberry, and hazelnut products, along with two nutraceutical formulations, were developed, characterized for bioactive content, and assessed for palatability, bioavailability, and safety. Adults aged ≥50 years participated in a 4-week intervention, consuming daily servings of functional foods and either a whey protein-based or essential amino acid-based nutraceutical. Compliance, acceptability, anthropometry, body composition, muscle strength, and biochemical markers were assessed pre- and post-intervention. **Results:** Functional foods and nutraceuticals were well-tolerated, with high adherence (>80%). Bioactive compounds were detectable in serum post-consumption, confirming bioavailability. Preliminary findings suggested that integrating functional foods and nutraceuticals into a Mediterranean-style dietary intervention is feasible, safe, and acceptable in older adults with metabolic risk factors. These results support the potential clinical benefit of combined dietary strategies and provide a rationale for a larger randomized controlled trial to evaluate efficacy on metabolic, musculoskeletal, and hepatic outcomes.

## 1. Introduction

Adequate nutrition is a cornerstone of public health, particularly in Western societies, where obesity and chronic degenerative diseases are increasingly prevalent [1,2]. A “healthy diet” should promote complete physical, mental, and social well-being, not merely the absence of disease [3]. This concept also embraces environmental sustainability, aiming to ensure food and nutrition security for both current and future generations. Sustainable diets are defined as nutritionally adequate, safe, culturally acceptable, economically fair, and environmentally low-impact, while preserving biodiversity and ecosystem integrity [3].

The global shift toward diets high in ultra-processed foods, refined sugars, saturated fats, and red meat, but poor in health-promoting nutrients, has contributed to the rise in overweight and obesity (affecting 2.1 billion people globally) [4,5], along with selective nutrient deficiencies. These dietary patterns are closely linked to the increasing incidence of non-communicable diseases (NCDs), including insulin resistance, hepatic steatosis, metabolic syndrome, sarcopenia, osteoporosis, type 2 diabetes, cardiovascular diseases, and certain cancers [2,6,7]. In Italy, approximately 24 million individuals are affected by chronic conditions, especially older adults (over 85% of those aged >75 years) and postmenopausal women [8,9]. If current trends persist, NCDs are expected to account for nearly two-thirds of the global disease burden by 2050 [10]. Unhealthy diets also significantly contribute to environmental degradation. The food system accounts for over 25% of global greenhouse gas (GHG) emissions, extensive water and marine pollution, and the use of nearly half of all ice-free land for agriculture [11,12]. With a projected 36% increase in global population by 2050, food-related GHG emissions could rise by 80%, reaching 4.1 gigatonnes of CO_2_-equivalent per year–an amount equivalent to emissions from the global transport sector in 2010 [13]. Agricultural expansion and rising food demand are also major drivers of deforestation and biodiversity loss.

Food waste further worsens this scenario. According to the FAO, one-third of global food production is lost or wasted across the supply chain [14]. In Italy, a significant proportion of waste occurs during primary production and industrial processing, particularly of fruits, vegetables, tubers (26%), and dairy products (21%), mainly due to inefficiencies and processing discards [14,15].

Addressing the interconnected challenges of diet, health, and environmental sustainability requires integrated strategies focused on reducing waste and ecological impact while promoting sustainable, health-enhancing lifestyles. Nutritional sustainability is grounded in biodiversity preservation, food security, waste reduction, and the functional quality of foods [16,17,18]. A global shift toward predominantly plant-based diets, such as Mediterranean, vegetarian, and pescatarian patterns, could mitigate the projected increase in food-related GHG emissions [19,20]. Nonetheless, a balanced inclusion of high-quality animal proteins may also be beneficial. When combined with whey proteins (WPs)—which are highly digestible and rich in branched-chain amino acids, these blends may enhance anabolic responses in older adults with sarcopenia, especially when co-supplemented with vitamin D, vitamin E, or L-carnitine. B vitamins, particularly B3, may further support muscle health by increasing NAD^+^ levels and promoting mitochondrial biogenesis in myopathic patients [21,22]. Branched-chain amino acids (BCAAs), and leucine in particular, serve as key nutrient signals activating the mTORC1 pathway; providing a leucine-rich amino acid load (~2–3 g leucine per feeding) is frequently cited as a threshold to maximally stimulate postprandial muscle protein synthesis in older adults, especially when total protein intake is marginal [23].

At the same time, increasing attention is being paid to nutraceuticals and functional foods, given their potential to prevent chronic diseases and mitigate the effects of industrial agriculture. Functional foods and nutraceuticals, those providing health benefits beyond basic nutrition, are now more accessible thanks to advances in food science and biotechnology [24,25]. In recent years, there has been a substantial increase in the consumption of functional foods and nutraceuticals, particularly for the prevention and management of metabolic and chronic diseases. In 2023, the global market was valued at approximately USD 317.22 billion, and it is projected to grow at a compound annual growth rate (CAGR) of 9.6% over the forecast period 2024–2030 [26]. The most prominent regional markets in this sector are North America, Europe, and the Asia-Pacific area [26].

Among these, several Mediterranean products rich in polyphenols show promising anti-inflammatory and antioxidant properties. *Citrus bergamia* (bergamot), endemic to Calabria, contains unique flavonoids with well-documented lipid-lowering and cardiometabolic effects. Its polyphenolic fraction (BPF) improves endothelial function and reduces serum cholesterol, low-density lipoprotein (LDL), triglycerides, and body weight in patients with metabolic syndrome [27,28,29,30]. Pomegranate, rich in cyanidin and delphinidin derivatives, offers antioxidant and anti-inflammatory potential relevant to age-related conditions [31]. Similarly, blueberries have shown benefits on glucose and lipid metabolism, as well as in reducing oxidative stress [32]. Hazelnuts, particularly those produced in regions such as Calabria (Southern Italy), are rich in monounsaturated fats, polyphenols, and bioactive compounds with proven cardiometabolic effects [33]. Lycopene, a carotenoid with high antioxidant capacity, especially abundant in ripe tomatoes grown in Southern Italy, has been associated with reduced all-cause and prostate cancer-specific mortality [34,35].

In this context, it becomes crucial to explore the clinical integration of sustainable, functional products as part of structured dietary interventions. While their biological plausibility and potential health benefits are supported by growing evidence, real-life data on their clinical feasibility, safety, palatability, and patient adherence remain limited. This feasibility study evaluated an innovative intervention combining functional foods and nutraceuticals with Mediterranean-style dietary counseling and lifestyle support in individuals with metabolic risk factors. The primary objectives were to assess feasibility, tolerability, and acceptability, while exploring preliminary effects on body composition, liver health, and bone status. Findings will inform the design of a future randomized controlled trial and guide the selection of clinically relevant outcomes and biomarkers.

## 2. Materials and Methods

### 2.1. Product Development and Testing

Prior to conducting this study in patients, all functional food prototypes underwent thorough characterization, palatability assessment, and bioavailability evaluation. Based on literature evidence and prior screening of bioactive compounds, prototypes were formulated for clinical evaluation. Specifically, the following products were selected for development and testing: a functional tomato sauce/juice rich in lycopene for bone health support [36,37]; a pomegranate and bergamot-based juice rich in antioxidants targeting bone health [29,38]; a bergamot marmalade rich in polyphenols to prevent metabolic disorders [39,40]; a Calabrian hazelnut cream and a blueberry juice, both rich in antioxidants and polyphenols, for metabolic health [41,42]; a nutraceutical formulation containing whey proteins, vitamins, minerals, and antioxidants for muscle health [43]; and a second nutraceutical based on essential amino acids, also aimed at improving muscle function [44]. Moreover, the bioactive compounds of each food ingredient used in the prototype were tested to assess their in vitro safety.

The functional food prototypes were developed in collaboration with CGF Foods, an agro-food company based in Calabria, while the nutraceutical formulations were produced by Enterfarma S.r.l. (Catania, Italy) and Errekappa Euroterapici S.r.l. (Milan, Italy), respectively. The nutritional characteristics of each functional product are reported in the Supplementary Table S1.

### 2.2. Phytochemical Characterization and Bioactive Compound Analysis of Functional Foods

All functional food prototypes, including Calabrian tomato sauce and juice, pomegranate, bergamot, and blueberry juices, bergamot marmalade, and hazelnut cream, underwent comprehensive phytochemical characterization to quantify their bioactive compound content and antioxidant activity. Standardized extraction and analytical protocols adapted from the scientific literature were applied according to the specific food matrix [45,46].

Carotenoids in tomato products were extracted by liquid–liquid extraction using organic solvents (hexane or tetrahydrofuran) under continuous stirring for 2 h in the dark to prevent degradation. Following centrifugation (1500 rpm, 5 min), the organic phase was collected and analyzed by High-Performance Liquid Chromatography (HPLC). An encapsulation formulation was prepared to evaluate carotenoid bioavailability in vitro.

Juices and marmalades were subjected to low-speed centrifugation (20× *g*) and filtration (0.22 µm) to remove solids prior to analysis. Total phenolic content (TPC) and total flavonoid content (TFC) were measured by Folin–Ciocalteu and aluminum chloride colorimetric assays, reading absorbance at 760 nm and approximately 360 nm, respectively. Gallic acid and a flavonoid standard mixture, containing rutin, naringin, hesperidin and apigenin in equal weight ratio, were used to prepare the calibration standard curves. TPC values were expressed in gallic acid equivalent (GAE). Flavonoid profiles were characterized by HPLC using a C18 column under gradient elution with acetonitrile and aqueous H_3_PO_4_ as mobile phases. Antioxidant capacity was assessed through the 2,2-Diphenyl-1-picrylhydrazyl (DPPH) radical scavenging assay, with L-ascorbic acid as positive control and inhibition percentage calculation, according to the literature protocol. In detail, 50 µL of sample were incubated in the dark for 30 min with 1 mL DPPH methanol solution (0.004% *w*/*w*). Methanol was used as blank, and 50 µL of ascorbic acid solution (2 mg/mL) were used as positive control, while DPPH pure solution represented the negative control. Absorbance at wavelength ~517 nm was obtained by a UV-vis spectrophotometer and inhibition percentage was calculated ac-cording to the following formula:

I (%) = [(A0 − A1)/A0] × 100

A0 = absorbance of negative control; A1 = absorbance of extracts/standards.

Due to the complex lipid matrix of the hazelnut cream, bioactives were extracted from freeze-crushed hazelnut powder by supercritical CO_2_ extraction with 20% ethanol co-solvent at 350 bar and 45 °C. Extracts were purified by centrifugation and filtration. TPC was quantified as above, and tocopherols were analyzed by HPLC on a C18 column using an acetonitrile: methanol (98:2) gradient at absorbance wavelengths of 210 and 270 nm. Antioxidant capacity was measured by the DPPH assay following the same procedure.

HPLC analyses of bioactive compounds were performed on a ThermoFisher Vanquish system (Thermo Fisher Scientific, Waltham, MA, USA) equipped with a UV/VIS variable wavelength detector and controlled by Chromeleon^®^ software (v7.2). Columns used included Acclaim^®^ 120 reverse phase C18 (5 µm, 100 mm × 4.6 mm) maintained at 25 °C. Mobile phase compositions and detection wavelengths varied by analyte: bergamot polyphenols were separated using an aqueous 10 mM H_3_PO_4_ and acetonitrile gradient (detection ~280 nm); hazelnut antioxidants utilized an acetonitrile/methanol (98:2) gradient (210–270 nm); vitamin B6 was analyzed with MilliQ water/acetonitrile (1:1) at 0.5 mL/min (210–290 nm). Injection volumes ranged from 10 to 20 µL, with run times between 10 and 65 min. Quantification relied on calibration curves prepared from standards (rutin, naringin, hesperidin, apigenin, α-tocopherol), all exhibiting linearity (r^2^ > 0.99).

This methodological approach enabled precise quantification of polyphenols, flavonoids, carotenoids, vitamins, and antioxidant activity across functional food matrices, providing the basis for subsequent bioavailability and efficacy assessments.

### 2.3. Cell Culture Conditions and Safety Evaluation of Functional Food-Derived Extracts

To evaluate the safety of extracts derived from functional foods, cell-based assays were conducted using in vitro models representative of bone, liver, and muscle tissues. Three cell lines were employed: MG-63 (human osteoblasts), HepG2 (human hepatocytes), and C2C12 (murine myoblasts). MG-63 and C2C12 cells were cultured in high-glucose Dulbecco’s Modified Eagle Medium (DMEM) supplemented with 10% fetal bovine serum (FBS) and 1% penicillin/streptomycin (P/S), while HepG2 cells were maintained in Minimum Essential Medium (MEM) enriched with essential amino acids, 10% FBS, and 1% P/S. C2C12 myoblasts were differentiated into myotubes for 12 days in DMEM containing 2% horse serum (HS). Cells were seeded in 96-well plates at a density of 1 × 10^4^ cells/well for MG-63 and HepG2, and 5 × 10^4^ cells/well for C2C12, and allowed to adhere for 48 h in complete culture medium. After adhesion, cells were treated for 24 h with various fruit-derived bioactive compounds or functional food extracts. All treatments were freshly prepared and directly diluted in serum-free culture medium to reach the desired final concentrations. MG-63 osteoblast-like cells were exposed to lycopene (1.25, 2.5, 5, and 10 μM) extracted from tomato sauce and encapsulated in 1,2-distearoyl-sn-glycero-3-phosphocholine liposome. Additional treatments included aqueous pomegranate extract (0.004, 0.04, 0.4, and 4 μg/mL), aqueous bergamot extract (0.001, 0.01, 0.1, and 1 μg/mL), or a combination of the two extracts.

HepG2 hepatocytes were treated individually with aqueous blueberry extract (3.75, 7.5, 15, and 30 µg/mL), a lipophilic hazelnut extract solubilized in ethanol (0.01, 0.1, and 1 µg/mL), or a bergamot mixture containing 95% pulp and 5% peel (0.01, 0.1, and 1 µg/mL).

C2C12 myotubes were treated with Nutraceutical 1 and Nutraceutical 2 at final concentrations of 0.125, 0.25, 0.5, and 1 mg/mL. In order to guarantee sterility, all extracts were subjected to 0.2 µm cellulose filtration before performing in vitro tests.

At the end of the treatment period, cell viability was assessed using the 3-(4,5-dimethylthiazol-2-yl)-2,5-diphenyltetrazolium bromide (MTT) assay. Briefly, a solution of MTT (Sigma, St. Louis, MO, USA) was added to each well to reach a final concentration of 5 mg/mL, followed by incubation at 37 °C for 2 h. The medium was then removed, and the resulting formazan crystals were solubilized in 100 µL of dimethyl sulfoxide (DMSO). Absorbance was measured at 570 nm, with background correction at 630 nm, using a microplate reader (BioTek 800 TS Absorbance Reader, BioTek Instruments, Inc., Winooski, VT, USA). For the proliferation assay, MG-63 cells treated with lycopene were detached, stained with trypan blue, and manually counted using the NucleoCounter NC-100 (ChemoMetec A/S, Lillerød, Denmark).

### 2.4. Sensory Evaluation and Palatability Assessment

Prior to inclusion in the feasibility study, all functional food and nutraceutical prototypes underwent sensory and palatability testing to evaluate consumer acceptability and organoleptic quality. Validated questionnaires tailored to each food matrix (sauce, juice, marmalade, cream, or powder) were administered via digital forms (Google Forms) (https://forms.gle/UqNCJeJCh9DNjdKXA, https://forms.gle/GZB1a85MrPoAkf386, https://forms.gle/naJ9nNAhQtzKTgkK7, both accessed on 25 March 2025).

The functional tomato sauce was evaluated at room temperature using a 9-point intensity scale (1 = very low/no intensity; 9 = maximum intensity) assessing consistency, flavor intensity, color, freshness, fruity notes, sweetness, acidity, bitterness, ripeness, tomato concentration, savoriness, astringency, and spiciness [47]. A functional tomato juice derived from the sauce was assessed similarly; participants consumed 200 mL and rated sensory attributes including color, aroma, sweetness, acidity, bitterness, astringency, viscosity, and overall acceptability on a 10-point scale [47].

Pomegranate, bergamot, and blueberry juices were evaluated following the same protocol: after consuming 200 mL, participants completed validated questionnaires rating chromatic intensity, transparency, aroma, freshness, sweetness, acidity, bitterness, viscosity, astringency, spiciness, persistence, attractiveness, and the intensity of pleasant/unpleasant odors.

The bergamot marmalade and hazelnut cream were assessed by both research staff and consumers using a 7-point hedonic scale (1 = dislike extremely; 7 = like extremely) for general acceptability, color, flavor, aroma, texture, taste, and spreadability. Participants consumed 30 g of each product and completed the corresponding questionnaires [48].

Two nutraceutical powder formulations were tested in individually packaged sachets, reconstituted with cold water. Sensory attributes (appearance, aroma, taste, texture, and overall acceptability) were rated using a 9-point hedonic scale [49]. Post-consumption questionnaires were completed to determine average scores and product acceptability.

### 2.5. Bioavailability

The bioavailability of functional molecules in various food matrices and nutraceutical formulations was evaluated in healthy human volunteers. Subjects (both sexes, aged 20–75 years) were recruited and instructed to follow a controlled dietary plan the day prior to product consumption, avoiding foods or supplements containing polyphenols (while being allowed to consume only fish, meat, rice, pasta, bread, and aged cheeses, and strictly avoiding tea, coffee, cocoa, chocolate, berries, fruit juices, nuts, legumes, spices, red wine, and any herbal or botanical supplements/extracts) or B-vitamins, depending on the tested product. For each intervention, fasting venous blood samples were collected at baseline and 120 min post-consumption of a defined dose of the functional product (30 g bergamot marmalade, 30 g hazelnut cream, 200 mL of blueberry juice, 200 mL of Calabrian tomato sauce or juice, 200 mL of pomegranate and bergamot juice, 25 g whey protein nutraceutical, or 11 g amino acid/vitamin nutraceutical) [50,51]. Blood samples were processed within one hour, centrifuged at 3500 rpm for 5 min at 4 °C to separate serum, which was then deproteinized by incubation with an equal volume of methanol, vortexed, and centrifuged again (21× *g*, 10 min, 4 °C). The resulting supernatant was analyzed by HPLC using a ThermoFisher Vanquish System equipped with a quaternary pump, split sampler, column compartment, and UV/VIS variable wavelength detector. Chromeleon^®^ software (v7.2) was used for data processing.

Columns employed included Acclaim^®^ 120 reverse phase C18 with a particle size of 5 µm, with the dimensions 100 mm × 4.6 mm, and maintained at 25 °C. Mobile phases varied according to the analyte: bergamot polyphenols were separated using an H_3_PO_4_ 10 mM aqueous solution and acetonitrile gradient with detection at ~280 nm; hazelnut antioxidants used an acetonitrile: methanol mixture (98:2) at flow rates of 0.3–1 mL/min with absorbance at 210–270 nm; vitamin B6 in nutraceuticals was analyzed with a mobile phase of MilliQ water and acetonitrile (1:1) at 0.5 mL/min and absorbance at 210–290 nm. Injection volumes ranged from 10 to 20 µL, with run times between 10 and 65 min. Quantification relied on calibration curves constructed from standards (e.g., rutin, naringin, hesperidin, apigenin for polyphenols; α-tocopherol for antioxidants; vitamin B6 standards), exhibiting linearity (r^2^ > 0.99) [52]. Total phenolic content in serum was additionally assessed via Folin–Ciocalteu colorimetric assay, expressed as mg gallic acid equivalents per mL serum. This comprehensive approach aimed to determine the extent to which bioactive compounds from these functional products are absorbed and detectable in human plasma, supporting their potential biological activity [53].

### 2.6. Subjects

The feasibility clinical study was conducted at the Clinical Nutrition Unit of the “R. Dulbecco” Azienda University Hospital of Catanzaro. The study protocol was approved by the Territorial Ethics Committee of the Calabria Region (Prot. CE n.74 of 18 March 2024) and conducted in accordance with the Declaration of Helsinki. The study comprised three phases: (1) screening, (2) intervention (Group 1 and 2), and (3) follow-up.

During screening, participants were evaluated for eligibility through medical examination. Subjects were recruited via public advertisements and selected based on predefined inclusion and exclusion criteria. Inclusion criteria were as follows: age ≥ 50 years; both sexes; apparently healthy; no dermatological or pharmacological treatments affecting the endpoints; provision of written informed consent in accordance with ethical guidelines. Exclusion criteria included the presence of renal or hepatic insufficiency, malignancies, malnutrition, or chronic obstructive pulmonary disease; concurrent participation in other clinical trials; dermatological conditions (e.g., eczema, acne, infections); known allergies to any ingredients in the test products; any other medical condition deemed unsuitable by the investigators; or inability to attend follow-up visits.

Participants with well-controlled chronic conditions (e.g., hypertension, dyslipidemia, type 2 diabetes, or osteoporosis) under stable pharmacological treatment were eligible for inclusion.

### 2.7. Study Design

The intervention period lasted a total of 8 weeks (4 weeks per group) and was conducted between April and May 2024. The intervention consisted of the daily consumption of a combination of functional foods and nutraceuticals developed specifically for this project. Participants were assigned to one of two groups, both receiving similar dietary components with the exception of the nutraceutical formulation.

Group 1

Participants consumed the following products daily:

- A total of 200 mL of Calabrian tomato sauce or juice naturally rich in lycopene;
- A total of 200 mL of blueberry juice (rich in antioxidants and polyphenols);
- A total of 200 mL of pomegranate and bergamot juice (antioxidant-rich);
- A total of 30 g of bergamot marmalade (high in polyphenols);
- A total of 30 g of hazelnut cream made with Calabrian hazelnuts (rich in antioxidants);
- A total of 50 g of Senatore Cappelli ancient grain pasta;
- A total of 1 sachet/day of Nutraceutical 2, containing whey proteins, vitamins, minerals, and antioxidants.

Group 2

Participants received the same products and dosages as Group 1, with the only difference being the substitution of the nutraceutical. Instead of Nutraceutical 2, they consumed the following:

- A total of 1 sachet/day of Nutraceutical 1, based on essential amino acids;
- The Senatore Cappelli pasta was consumed four times per week.

All products were provided weekly, and participants received detailed instructions on their correct usage. Compliance was monitored through daily intake diaries completed by participants and reviewed weekly by the study team.

Additionally, all participants received personalized dietary counseling and nutritional recommendations based on the Mediterranean Diet, aimed at promoting long-term adherence to healthy eating patterns throughout the intervention period.

Anthropometry, body composition, handgrip strength, transient elastography, quantitative ultrasound were performed in the morning following a 12 h overnight fast. Participants were instructed to abstain from caffeine, stimulants, and smoking prior to assessments. Usual pharmacological therapies were maintained, as they did not interfere with fasting measurements.

### 2.8. Adherence of Treatments

Treatment adherence was assessed through structured patient interviews combined with product accountability. For each functional food tested (sachets, beverages, pasta, and jam), the number of unused portions returned at study visits was recorded and compared with the expected intake, thereby providing an estimate of compliance with the prescribed intervention. Participants were classified as low adherent if they consumed less than 80% of the prescribed treatment.

### 2.9. Anthropometry and Body Composition

Body weight and height were measured using calibrated instruments, and the body mass index (BMI) was calculated as weight (kg) divided by height squared (m^2^). Waist and hip circumferences were measured with a non-stretchable tape measure, and waist-to-hip ratio (WHR) was derived. Mid-arm circumference was measured at the midpoint between the acromion and the radial bone. Triceps skinfold thickness was assessed three times using a GIMA caliper, and the mean value was recorded.

Body composition was assessed via bioelectrical impedance analysis (BIA 101 RJL/Akern; Detroit, MI, USA/Firenze, Italy) with a tetrapolar hand-to-foot electrode configuration. Resistance (R), reactance (Xc) was measured. BodyGram Plus software Version 3.0.33 (Akern; Firenze, Italy) was used to estimate fat-free mass (FFM), fat mass (FM), skeletal muscle mass (SMM), and appendicular skeletal muscle mass (ASMM). Measurements followed standardized protocols, and all assessments were performed by the same trained operator.

### 2.10. Muscle Strength and Sarcopenia

Handgrip strength (HGS) was measured three times per hand using a digital dynamometer (GRIPWISE, Matosinhos, Portugal), and the mean value was used for analyses. Sarcopenia was diagnosed according to EWGSOP2 criteria [54]: HGS < 27 kg for men and <16 kg for women, combined with ASMM < 20 kg for men and <15 kg for women.

### 2.11. Assessment of the Hepatic Parenchyma

Liver fat and stiffness were assessed via transient elastography (Fibroscan^®^ EXPERT 630; Echosense, Paris, France) using the M probe. Controlled attenuation parameter (CAP, dB/m) and liver stiffness (kPa) were recorded. Valid measurements required ≥10 successful acquisitions with interquartile range/median ratio (IQR/M) < 30%. Steatosis was graded as S1 (247–268 dB/m), S2 (269–280 dB/m), and S3 (≥281 dB/m) [55]. All assessments were performed by the same experienced operator.

### 2.12. Assessment of Bone Health Status

Calcaneal bone status was evaluated using quantitative ultrasound (QUS) (Sonost-3000; Osteosys, Seoul, Republic of Korea). Speed of sound (SOS) and broadband ultrasound attenuation (BUA) were measured. T-scores were classified as normal (>−1), osteopenia (−1 to −2.69), and osteoporosis (≤−2.7) [56]. In cases of prior fractures, the contralateral heel was assessed.

### 2.13. Adverse Events

Adverse events (AEs) were monitored using patient-reported questionnaires designed to capture new symptoms, type, and the severity of AEs potentially related to the intervention.

### 2.14. Statistical Analysis

All collected data were analyzed. Continuous variables were expressed as mean ± standard deviation (SD), while categorical data were reported as percentages (%). Within-group differences between baseline and follow-up values were evaluated using the two-tailed paired Student’s *t*-test. A *p*-value of < 0.05 was considered statistically significant. Statistical analyses were performed using SPSS version 29.0 for Windows (IBM Corp., Armonk, NY, USA).

## 3. Results

### 3.1. Carotenoids Quantification and Formulation from Tomato Juice and Sauce

**Figure 1:**
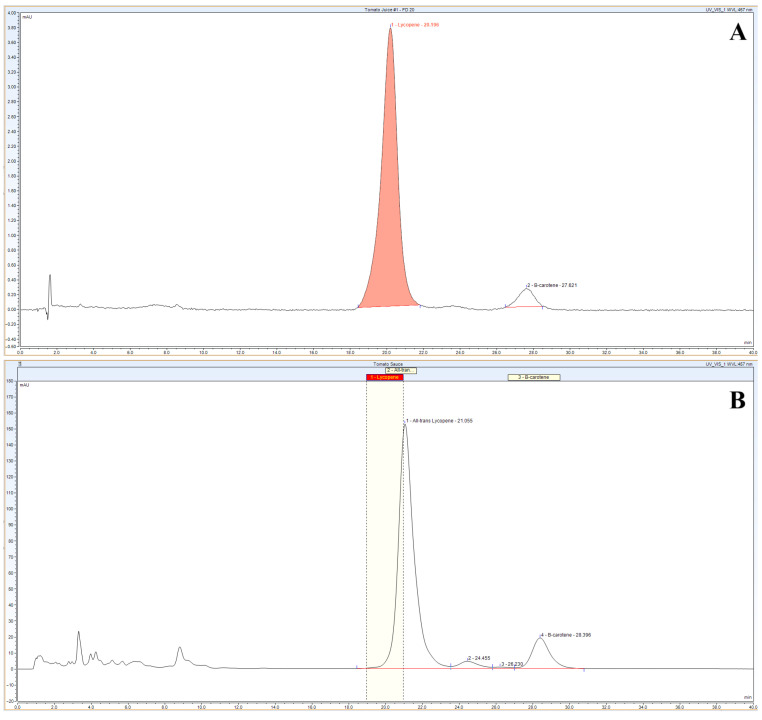
HPLC analyses of carotenoids extracted from tomato juice (**A**) and tomato sauce (**B**). Lycopene and β-carotene were separated and quantified at 457 nm in C18 reverse phase column.

HPLC analysis (C18 column, detection at 457 nm) quantified 55.53 ± 0.43 mg/100 g of lycopene and 22.89 ± 0.16 mg/100 g of β-carotene in tomato juice (Figure 1A) and 24.75 ± 0.31 mg/100 g and 7.83 ± 0.43 mg/100 g, respectively, in tomato sauce (Figure 1B), with minor differences attributed to matrix effects.

Overall, Calabrian tomato derivatives from “on-the-vine” ripening are confirmed as a rich and bioavailable source of carotenoids, suitable for cellular applications.

### 3.2. Polyphenol and Flavonoid Profiles and Antioxidant Capacity of Bergamot, Pomegranate, Blueberry, and Hazelnut Extracts

Pomegranate and bergamot juices exhibited a total phenolic content of 0.478 ± 0.010 mg/mL GAE (Table 1) and a total flavonoid content of 1.16 ± 0.03 mg/mL.

**Figure 2:**
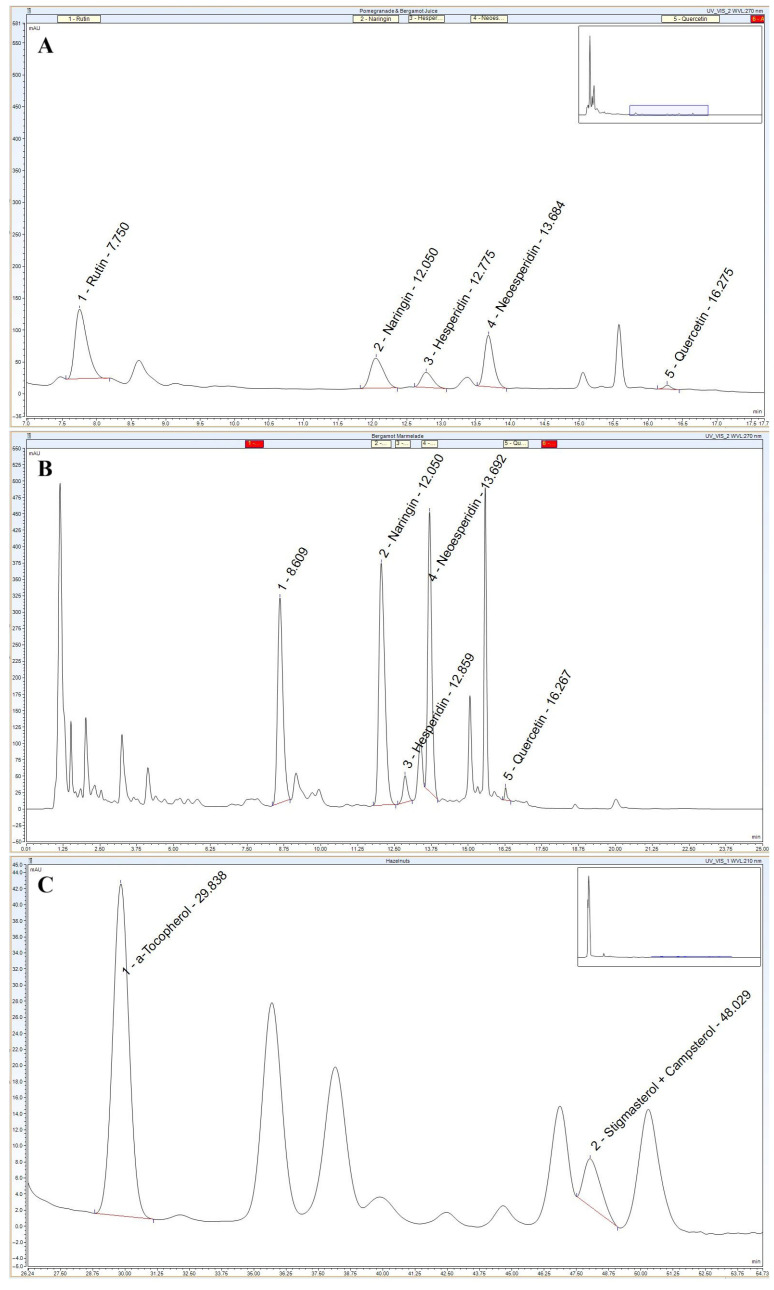
HPLC analyses of pomegranate and bergamot juice (**A**), bergamot marmalade (**B**) and Hazelnut CO_2_ extract (**C**).

HPLC analysis identified 12 flavonoid compounds, with naringin (0.043 ± 0.001 mg/mL; 42.85 ± 1.22 ppm), hesperidin (0.037 ± 0.001 mg/mL; 37.29 ± 1.26 ppm), and rutin (0.011 ± 0.0001 mg/mL; 11.48 ± 0.38 ppm) as the most abundant (Figure 2A). Antioxidant activity revealed 53.91 ± 1.23% inhibition, comparable to the ascorbic acid standard (Table 1), confirming the high antioxidant potential of these juices and their suitability for cellular applications.

Similarly, in a hydrophilic solution of bergamot marmalade (0.5 g/mL), total phenolic and flavonoid contents were 0.503 ± 0.065 mg/mL GAE (Table 1) and 0.657 ± 0.001 mg/mL (657.07 ± 0.5 ppm), respectively. HPLC analysis showed hesperidin 0.174 ± 0.019 mg/mL (174.25 ± 18.74 ppm) as the main flavonoid, followed by naringin at 0.009 ± 0.0001 mg/mL (8.9 ± 0.5 ppm) (Figure 2B). DPPH assay revealed 39.33 ± 0.37% inhibition (Table 1), indicating substantial antioxidant activity.

Blueberry juice showed a total phenolic content of 0.586 ± 0.015 mg/mL GAE (Table 1) and a flavonoid content of 0.886 ± 0.098 mg/mL, with moderate antioxidant activity (30.95 ± 3.53% inhibition) (Table 1).

Finally, the hazelnut CO_2_ extract had a total phenolic content of 0.228 ± 0.012 mg/mL GAE (Table 1), and α-tocopherol was identified as the main bioactive compound (0.009 ± 0.001 mg/mL; 9.08 ± 1.38 ppm) (Figure 2C). Its antioxidant activity, evaluated by DPPH, was 26.96 ± 0.75% (Table 1), indicating a moderate radical scavenging capacity.

### 3.3. Food-Derived Extracts Show a Safe Profile In Vitro

**Figure 3:**
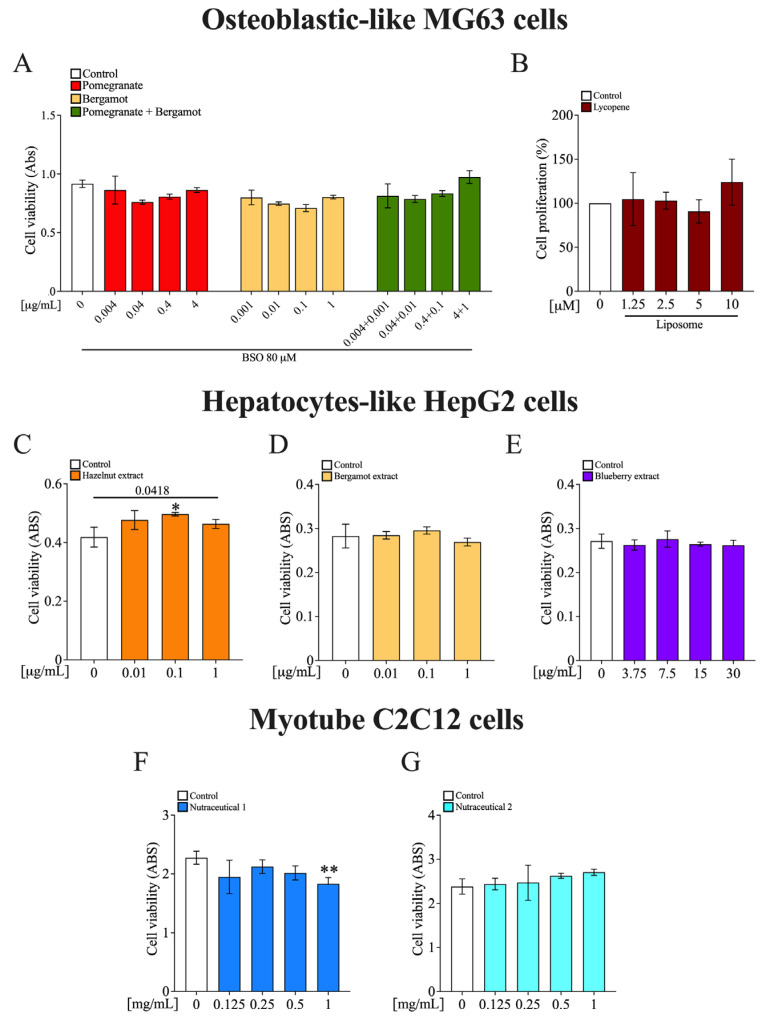
Food-derived extracts are safe for MG-63, HepG2 and C2C12 cell lines. (**A**) MG-63 osteoblasts were treated with pomegranate (0.004–4 µg/mL), bergamot (0.001–1 µg/mL), or their combination in the presence of BSO (80 µM). Cell viability was assessed by MTT assay. (**B**) Cell proliferation in MG-63 cells following treatment with liposomal lycopene (1.25–10 µM) was measured by direct cell count. (**C**–**E**) HepG2 were treated with hazelnut extract (0.01–1 µg/mL), bergamot extract (0.01–1 µg/mL), and blueberry extract (3.75–30 µg/mL), and cell viability was assessed by MTT assay. (**F**,**G**) C2C12 myotubes were treated for 48 h with increasing concentrations of two different nutraceutical formulations (0.125–1 mg/mL), and cell viability was measured via MTT assay. Data are represented as mean ± SD of three independent experiments and *p*-values are calculated by Student’s *t*-test: * *p* < 0.05, ** *p* < 0.01 vs. control.

To evaluate the safety of the functional foods, the extracts were tested on different cell lines representing bone, liver, and muscle. In bone cells, pomegranate and bergamot extracts, either alone or in combination, did not produce cytotoxic effects (Figure 3A). Similarly, lycopene encapsulated in liposomes did not alter bone cell proliferation at any of the tested concentrations (Figure 3B). Next, in liver cells, hazelnut extract significantly increased cell viability at 0.1 µg/mL compared to the control (*p* < 0.05; Figure 3C), whereas bergamot and blueberry extracts did not induce significant changes in viability (Figure 3D,E). Finally, in muscle cells, nutraceutical formulation 1 caused a significant reduction in cell viability at 1 mg/mL (*p* < 0.01; Figure 3F), while nutraceutical formulation 2 showed no effects at any of the concentrations tested (Figure 3G). Overall, these results suggest that the tested extracts are generally safe across different cell types.

### 3.4. Palatability and Bioavailability Assessments

All functional products were well tolerated and showed excellent sensory acceptability in healthy volunteers. Tomato sauce (2 women, 1 man; age 34 ± 5 years, BMI 21.9 ± 1 kg/m^2^) and tomato juice (3 women; age 36 ± 10 years, BMI 21.4 ± 2 kg/m^2^) received high scores for color, flavor, freshness, and maturity, and daily intake of 200 g for three days led to a significant increase in plasma lycopene levels, from undetectable baseline to 1.26 ± 0.04 µM (sauce) and 4.89 ± 0.33 ppm (0.005 ± 0.0001 mg/mL juice), confirming high bioavailability of carotenoids naturally enriched via “ripening on the vine” agronomy. Pomegranate and bergamot juice (2 men, 1 woman; age 36 ± 3 years, BMI 27.5 ± 5 kg/m^2^) was also highly accepted and well tolerated; its sensory profile reflected the acidity of bergamot and absence of added sugars. Serum analysis at 120 min post-consumption revealed a significant rise in hesperidin levels from baseline (mostly undetectable) to 5.35 ± 2.71 ppm (0.005 ± 0.003 mg/mL), indicating efficient polyphenol absorption. Similarly, bergamot marmalade (2 women, 1 man; age 40 ± 9 years, BMI 24.7 ± 4 kg/m^2^) was well tolerated and increased serum hesperidin to 7.86 ± 3.08 ppm (0.008 ± 0.003 mg/mL) at 120 min, supporting its systemic bioavailability.

Calabrian hazelnut cream (2 men, 1 woman; age 36 ± 3 years, BMI 27.5 ± 5 kg/m^2^) showed high acceptability for taste, aroma, and texture; serum α-tocopherol increased to 10 ± 12 ppm (0.01 ± 0.01 mg/mL) at 120 min, confirming efficient absorption. Blueberry juice (3 women; age 29 ± 1 years, BMI 23.9 ± 4 kg/m^2^) was well accepted and led to a significant increase in serum total phenolics (0.094 ± 0.019 ppm at 120 min). Nutraceutical 1 (3 women; age 35 ± 11 years, BMI 25.6 ± 5 kg/m^2^) containing whey proteins, vitamins, minerals, and antioxidants, and Nutraceutical 2 (two groups of 3 women each) containing amino acids and vitamins were both highly palatable and free from adverse effects. Notably, Nutraceutical 2 significantly increased serum vitamin B6 levels by 58% (14.45 ± 3.62 ppm; 0.014 ± 0.004 mg/mL) and 43% (9.10 ± 6.4 ppm; 0.009 ± 0.006 mg/mL) at 120 min in the two groups, respectively. Collectively, these findings, supported by in vitro safety assays, confirm the excellent acceptability, safety, and systemic bioavailability of all tested functional foods and nutraceuticals, supporting their suitability for clinical application.

### 3.5. Clinical Characteristics of Participants According to the Treatments

The feasibility study enrolled a cohort of 19 participants (mean age 66 ± 6 years; BMI 29.1 ± 4 kg/m^2^) selected according to predefined inclusion and exclusion criteria (see Section 2 and Table 2).

Over the 4-week intervention period, which combined innovative, environmentally sustainable functional foods and nutraceuticals with Mediterranean-style dietary guidance and lifestyle modifications, no adverse events or dropouts were reported. Adherence to all functional foods and nutraceutical products exceeded 90%, indicating excellent participant compliance and high acceptability of the intervention (see Table 3).

Despite the relatively short duration, clinically relevant improvements were observed in several health parameters. Mid-arm circumference increased significantly by 2.2% (*p* = 0.02), while triceps skinfold thickness decreased by 11% (*p* = 0.02), suggesting increased muscle mass and reduced subcutaneous fat. Total body water percentage also rose significantly (*p* = 0.02), reflecting improved hydration status. Bone health assessments demonstrated positive changes, with an 11% increase in the broadband ultrasound attenuation (BUA) T-score at the calcaneus (*p* = 0.04), indicative of enhanced bone quality (Table 4).

**Figure 4:**
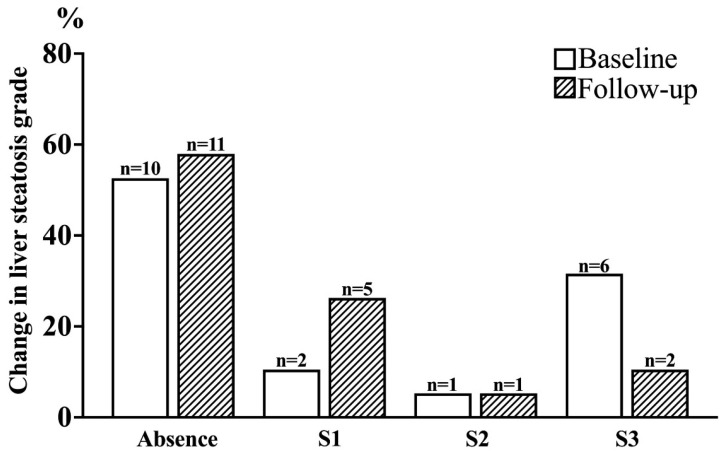
Change in Liver Steatosis grade at baseline and after 4 weeks of treatment with functional foods and nutraceuticals.

Liver evaluations revealed a 21% reduction in the number of participants with severe hepatic steatosis (S3 grade), a 5% increase in steatosis-free individuals, and a 16% increase in those with mild steatosis (S1 grade) (Figure 4).

Overall, 26% of participants exhibited an improvement in liver steatosis grade following the intervention, with no cases of worsening steatosis detected.

## 4. Discussion

This clinical study offers preliminary but robust evidence on the feasibility, safety, and potential biological efficacy of an integrated nutritional-lifestyle intervention combining sustainably sourced functional foods and nutraceuticals with a Mediterranean dietary approach. Leveraging a multidimensional methodology, from phytochemical profiling to clinical readouts, this work bridges basic nutritional biochemistry with translational and ecological health paradigms. The functional matrices under investigation were selected not only for their traditional use and territorial identity but also for their scientifically substantiated bioactive profiles. Analytical data confirmed that the Calabrian tomato sauce and juice were exceptionally rich in carotenoids, particularly lycopene (up to 64 ± 5 mg/100 g), with values exceeding those reported in many commercial preparations or tomato-based nutraceuticals [57,58,59]. Lycopene is a well-characterized antioxidant with emerging roles in inflammation modulation, lipid metabolism, and prostate health [60,61], and its significant increase in plasma levels after short-term intake underscores both the bioavailability of the matrix and the potential clinical relevance.

Similarly, the pomegranate and bergamot juices, as well as bergamot marmalade, demonstrated high contents of polyphenols and flavonoids, such as hesperidin, naringin, and anthocyanins, known to exert hepatoprotective, vasoprotective, and anti-inflammatory activities [27,28,62]. Notably, the presence of these compounds was confirmed via HPLC, and their absorption was supported by serum analyses showing rapid postprandial increases. These findings align with previous evidence indicating that polyphenols and flavonoids from citrus fruits and other botanical sources may beneficially modulate hepatic lipid metabolism and attenuate liver steatosis, primarily through activation of AMP-activated protein kinase (AMPK) and inhibition of nuclear factor kappa B (NF-κB) signaling pathways [34,50,63,64].

Furthermore, the inclusion of a Calabrian hazelnut extract, naturally enriched in α-tocopherol, contributed additional antioxidant and lipid-modulating effects. The detection of increased serum α-tocopherol levels following consumption suggests efficient systemic delivery, an aspect often limited in lipid-rich matrices [65,66]. The blueberry juice, still demonstrated moderate antioxidant activity and satisfactory bioavailability, potentially owing to its high anthocyanin content, which has been implicated in cardiometabolic and cognitive protection [32].

From a sensory and acceptability perspective, all tested products received consistently high scores, which is crucial for ensuring adherence in real-world dietary interventions. Reflecting this, the study demonstrated excellent compliance with no participant dropouts or adverse events reported throughout the intervention. Unlike many functional formulations characterized by poor palatability or requiring pharmacological administration [67], these food-based products were seamlessly integrated into participants’ daily routines, highlighting their strong potential for practical application.

The clinical phase of the study, despite its exploratory nature and limited sample size (*n* = 19), revealed meaningful trends across multiple endpoints. The significant increase in mid-arm circumference, coupled with reduced triceps skinfold thickness and enhanced total body water, may indicate a favorable shift in body composition, occurring without significant changes or reductions in overall body weight.

These effects are likely attributable to the synergistic role of high-quality proteins (e.g., whey), essential amino acids, and bioavailable micronutrients such as B-vitamins, which support protein synthesis, muscle function, and hydration [21,22,68,69].

Bone health improvement, reflected in an 11% increase in BUA T-score, aligns with the literature indicating that not only calcium and vitamin D but also vitamin K, polyphenols, and certain amino acids can positively influence bone remodeling [70].

The hepatic outcomes are particularly noteworthy: a reduction in the prevalence of severe steatosis (S3) and an overall improvement in liver fat grade in 26% of participants, as assessed via transient elastography. These results support the hypothesis that bioactive-rich food matrices, especially those high in flavonoids and polyphenols, may ameliorate liver fat accumulation and associated metabolic dysfunction [71,72]. Such findings are of particular relevance given the rising prevalence of liver steatosis, for which effective, non-pharmacological interventions remain limited.

A central strength of this study lies in its alignment with the principles of sustainable nutrition and planetary health. The intervention utilized minimally processed, biodiversity-preserving food sources such as bergamot, Calabrian tomatoes, and hazelnuts, ingredients endemic to the Mediterranean basin and traditionally underutilized in standardized clinical nutrition. This approach reflects the One Health perspective, recognizing the interconnectedness of human, environmental, and agricultural health [73,74].

However, several limitations warrant careful consideration. This study lacked a placebo-controlled arm, and the intervention duration (four weeks) was insufficient to assess long-term outcomes or sustainability of the observed effects. Bone density was assessed using quantitative ultrasound rather than dual-energy X-ray absorptiometry (DXA), limiting comparability with gold-standard data. Additionally, the small sample size restricted statistical power and precluded stratified analyses by sex, age, or metabolic phenotype. Moreover, in this study we used cancer-derived and highly proliferative cell lines, which do not fully reflect the physiology of normal bone and liver; therefore, the in vitro findings should be interpreted with caution. These limitations were intrinsic to the study’s phase I exploratory nature, aimed primarily at feasibility and biological signal detection.

Looking ahead, the encouraging results reported here form the basis for a planned randomized, controlled trial with larger cohorts, extended intervention duration, and chemical and in vitro characterization. These future studies will integrate inflammatory, oxidative, and metabolic markers, to uncover mechanisms of action and inter-individual variability in response to dietary bioactives. Particular attention will also be paid to gender differences in nutritional response, in line with the emerging field of precision nutrition and nutrigenomics [75,76,77].

In summary, this study provides evidence supporting the hypothesis that an innovative nutritional approach, based on the daily consumption of a combination of functional foods and nutraceuticals derived from sustainable agro-ecological systems, can effectively enhance metabolic, hepatic, and musculoskeletal health. When carefully formulated and integrated within a structured dietary regimen, these products not only exert individual bioactive effects but also represent a novel paradigm in clinical nutrition that simultaneously advances health promotion and environmental sustainability. The findings confirm the feasibility, safety, and preliminary efficacy of this multi-component intervention combined with lifestyle modifications. Observed improvements in body composition, bone density, and liver function underscore the potential of this integrated strategy to improve overall health outcomes, quality of life, and well-being in the target population. Taken together, these results highlight the promising role of sustainably sourced, well-formulated functional foods and nutraceuticals as key components of ecologically responsible nutritional strategies aimed at promoting long-term health.

## 5. Conclusions

This feasibility study provides preliminary yet compelling evidence that the integration of sustainable functional foods and nutraceuticals, derived from Mediterranean biodiversity, can be safely and effectively incorporated into clinical dietary protocols. The intervention demonstrated good tolerability, high palatability, and encouraging trends toward improvement in metabolic, hepatic, and skeletal health parameters, even over a relatively short observation period.

Beyond clinical outcomes, this study highlights a translational model bridging nutritional science, sustainability, and cultural heritage. By valorizing local agro-biodiversity and minimizing environmental impact, this approach aligns with the principles of planetary health and the One Health paradigm. The successful formulation, characterization, and bioavailability of selected bioactive compounds reinforce the feasibility of using food-derived bioactives in precision nutrition strategies.

Overall, these findings support the potential of eco-innovative, patient-centered nutrition to address the dual challenges of chronic disease prevention and environmental preservation. Functional foods, when rooted in scientific evidence and local tradition, represent a valuable ally in building resilient, health-promoting food systems for the future.

## Figures and Tables

**Table 1:** Chemical Characterization.

**Sample Name**	**TPC** **(GAE mg/mL ± SD)**	**TFC** **(mg/mL ± SD)**
Pomegranate and bergamot juice	0.478 ± 0.010	1.16 ± 0.03 mg/mL
Bergamot marmalade	0.503 ± 0.065	0.657 ± 0.001 mg/mL
Blueberry juice	0.586 ± 0.015	0.886 ± 0.098 mg/mL
Hazelnuts *	0.228 ± 0.012	-
**Antioxidant power of functional food**		
**Sample**	**I (%) ± SD**	
Pomegranate and bergamot juice	53.91 ± 1.23	
Bergamot marmalade	39.33 ± 0.37	
Blueberry juice	0.586 ± 0.015	
Hazelnuts *	26.96 ± 0.75	

* CO_2_ extract; GAE = gallic acid equivalents; I (%): percentage of inhibition; SD = standard deviation. All values are expressed as mean ± SD of three independent measurements.

**Table 2:** Baseline demographic and clinical characteristics of participants.

Variables	Population (*n* = 19)
Age (years)	66 ± 6
Weight (kg)	77 ± 13
BMI (kg/m^2^)	29.1 ± 4.0
WC (cm)	99 ± 13
HC (cm)	107 ± 9
WHR	0.93 ± 0.1
Mid-arm (cm) circumference	32 ± 3
Triceps skinfold (mm)	2.6 ± 1.0
Handgrip (kg)	27.1 ± 8.0
**Body composition**	
TBW (%)	50 ± 7
FFM (%)	68 ± 10
FM (%)	32 ± 10
SMM (kg)	33 ± 7
ASMM (kg)	19 ± 4
RMR (kcal)	1533 ± 172
BUA BMD (g/cm^2^)	0.439 ± 0.1
BUA T-score (SD)	−1.27 ± 1.0
CAP score (dB/m)	245 ± 45
CAP score SD (%)	10 ± 5
Stiffness (kPa)	4.8 ± 1.0
Stiffness IQR	15 ± 6
**Prevalence**	
Gender (Female, %)	68
Obesity (%)	47
Low Handgrip (%)	0
Sarcopenia (%)	0
Osteopenia (%)	37
Osteoporosis (%)	16
Liver steatosis S0 (%)	53
Liver steatosis S1 (%)	11
Liver steatosis S2 (%)	5
Liver steatosis S3 (%)	32
Liver Fibrosis (%)	0

Abbreviations: BMI = body mass index, WC = waist circumference, HC = hip circumference, WHR = waist to hip ratio, TBW = total body water, FFM = free fat mass, FM = fat mass, SMM = skeletal mass muscle, ASMM = appendicular skeletal mass muscle, RMR = resting metabolic rate, BUA = broadband ultrasound attenuation; BMD = bone mineral density; CAP = controlled attenuation parameter, SD = standard deviation, IQR = interquartile range.

**Table 3:** Adherence to all functional foods and nutraceutical products.

Functional Products	Adherence to Treatment (%)
Tomato sauce/tomato juice naturally rich in lycopene	92
Blueberry juice rich in antioxidants and polyphenols	96
Pomegranate and bergamot juice rich in antioxidants	95
Bergamot marmalade rich in antioxidants and polyphenols	94
Hazelnut cream rich in antioxidants and polyphenols	92
Nutraceutical 1 with essential amino acids	94
Nutraceutical 2 with whey proteins, vitamins, minerals, and antioxidants	96

**Table 4:** Baseline and follow-up clinical characteristics of all participants.

Variables	Basal	Follow-Up	*p*-Value
Weight (kg)	77 ± 13	77 ± 13	0.77
BMI (kg/m^2^)	29.1 ± 4.0	29.2 ± 4.0	0.60
WC (cm)	99 ± 13	101 ± 13	0.07
HC (cm)	107 ± 9	108 ± 9	0.12
WHR	0.93 ± 0.1	0.93 ± 0.1	0.42
Mid-arm circumference (cm)	32 ± 3	33 ± 4	0.020
Triceps skinfold (mm)	2.6 ± 1.0	2.3 ± 1.0	0.021
Handgrip (kg)	27.1 ± 8.0	27.8 ± 7.0	0.14
*Body composition*			
TBW (%)	50 ± 7	51 ± 8	0.023
FFM (%)	68 ± 10	68 ± 9	0.60
FM (%)	32 ± 10	32 ± 9	0.60
SMM (kg)	33 ± 7	33 ± 7	0.12
ASMM (kg)	19 ± 4	20 ± 4	0.20
RMR (kcal)	1533 ± 172	1512 ± 181	0.08
BUA BMD (g/cm^2^)	0.439 ± 0.1	0.452 ± 0.1	0.733
BUA T-score (SD)	−1.27 ± 1.0	−1.14 ± 0.9	0.042
CAP score (dB/m)	245 ± 45	234 ± 43	0.12
Stiffness (kPa)	4.8 ± 1.0	4.6 ± 1.0	0.60

Abbreviations: BMI = body mass index, WC = waist circumference, HC = hip circumference, WHR = waist to hip ratio, TBW = total body water, FFM = free fat mass, FM = fat mass, SMM = skeletal mass muscle, ASMM = appendicular skeletal mass muscle, RMR = resting metabolic rate, BUA = broadband ultrasound attenuation; BMD = bone mineral density; CAP = controlled attenuation parameter.

## Data Availability

The data presented in this study are available on request from thecorresponding author. Due to the project agreements and collaborations with commercial partners, the distribution of the data must be monitored and approved on a case-by-case basis to ensure that their use remains consistent with the defined objectives. Therefore, the dataset is available only upon request to the corresponding author.

## Supplementary Materials

The following supporting information can be downloaded at https://www.mdpi.com/article/10.3390/nu17243858/s1, Table S1. Composition of functional foods and nutraceutics.

## Abbreviations

The following abbreviations are used in this manuscript:

ASMM	Appendicular Skeletal Muscle Mass
BCAAs	Branched-Chain Amino Acids
BIA	Bioelectrical Impedance Analysis
BMD	Bone Mineral Density
BMI	Body Mass Index
BPF	Bergamot Polyphenolic Fraction
BUA	Broadband Ultrasound Attenuation
CAP	Controlled Attenuation Parameter
CAGR	Compound Annual Growth Rate
CO_2_-Ceq	Carbon Dioxide–Equivalent
DMSO	Dimethyl Sulfoxide
DMEM	Dulbecco’s Modified Eagle Medium
DPPH	2,2-Diphenyl-1-picrylhydrazyl
EWGSOP2	European Working Group on Sarcopenia in Older People 2
FBS	Fetal Bovine Serum
FFM	Fat-Free Mass / Free Fat Mass
FM	Fat Mass
GAE	Gallic Acid Equivalent
GHG	Greenhouse Gas
HC	Hip Circumference
HGS	Handgrip Strength
HPLC	High-Performance Liquid Chromatography
HS	Horse Serum
IQR	Interquartile Range
IQR/M	Interquartile Range to Median Ratio
LDL	Low-Density Lipoprotein
MEM	Minimum Essential Medium
MTT	3-(4,5-Dimethylthiazol-2-yl)-2,5-diphenyltetrazolium bromide
NAD^+^	Nicotinamide Adenine Dinucleotide (oxidized form)
NCDs	Non-Communicable Diseases
P/S	Penicillin/Streptomycin
QUS	Quantitative Ultrasound
R	Resistance
RMR	Resting Metabolic Rate
SD	Standard Deviation
SMM	Skeletal Muscle Mass
SOS	Speed of Sound
TBW	Total Body Water
TFC	Total Flavonoid Content
TPC	Total Phenolic Content
UV-Vis	Ultraviolet–Visible (Spectrophotometry)
WC	Waist Circumference
WHR	Waist-to-Hip Ratio
WP/WPs	Whey Proteins
Xc	Reactance
