# Supplementary material for: Clinical Implementation of Sustainable Functional Foods and Nutraceuticals in Metabolic Health: A Feasibility Study
Source: Nutrients. 2025 Dec 10;17(24):3858. doi: 10.3390/nu17243858 (PMC12736368; doi:10.3390/nu17243858)
Supplement: Supplementary file 1 [file nutrients-17-03858-s001.zip › nutrients-4003825-supplementary.pdf]

Supplementary Materials

Supplementary Table S1. Composition of functional foods and nutraceutics.

Food	Ingredients	Daily amount	Bioactive compound	Expected physiological function
Organic Blueberry Nectar	Blueberries*, water, grape sugar*, lemon juice*, elder juice*, antioxidant: L-ascorbic acid.<br>Energy per 100 g: 78 kcal<br>Total Carbohydrate: 18 g.	200 ml	Antioxidants	Prevention of metabolic disorders in adults
Organic Pomegranate and Bergamot Nectar	Pomegranate juice* (40%), water, grape sugar*, bergamot juice from concentrated* (10%), elder juice*, gelling agent: fruit pectin, antioxidant: L-ascorbic acid.<br>Energy per 100 g: 45 kcal<br>Total Carbohydrate: 19 g.	200 ml	Antioxidants	Improvement of bone health in adults
Organic Bergamot jam	Bergamots*, grape sugar*, gelling agent: locust bean gum*. Fruit used: 60 g per 100 g; total sugars: 35 g per 100 g.	30 g	Antioxidants	Prevention of metabolic diseases in adults
Spreadable Cream with Hazelnuts from Calabria	Calabrian nuts (40%), sugar (32%), sunflower oil, butter, cocoa (0.5%), salt.<br>Total Energy per 100 g: 239 kcal<br>Total Carbohydrate: 1.9 g.	30 g	Fatty acids, sterols, antioxidants	Prevention of metabolic diseases in adults
Calabrian Tomato sauce or juice	Tomatoes*, lemon juice*, salt.<br>Energy per 100 g: 28 kcal<br>Total Carbohydrate: 3.6 g.	200 ml	Antioxidants	Improvement of bone health in adults
Pasta Senatore Cappelli with ancient grains	Energy per 100 g: 354 kcal<br>Total Carbohydrate: 72 g<br>Proteins: 12.5 g<br>Fibers: 7 g	50 g	Fibers	Improvement of gastrointestinal health in adults
**Nutraceutics**				
Nutraceutic n.1	Whey protein, potassium citrate, inulin, magnesium citrate, taurine, acetyl-L-carnitine hydrochloride, vitamin B3 (niacin), vitamin B5 (calcium pantothenate), folic acid, vitamin B6 (pyridoxine hydrochloride); flavoring: cocoa, vanilla. Emulsifier: xanthan gum. Sweeteners: sucralose, acesulfame K. Gluten-	1 sachet	Whey proteins, vitamins, minerals, antioxidants	Improvement of bone and muscle health in adults

	free. The nutraceutical provides, per 25 g of product, approximately: Protein: 15 g; Potassium: 540 mg; Taurine and L-carnitine: 0.3 g<br>Energy: 80 kcal per 25 g of product.			
Nutraceutic n.2	L-Leucine, L-Lysine, L-Isoleucine, L-Valine, L-Threonine, L-Cystine, L-Histidine, L-Phenylalanine, L-Methionine, L-Tyrosine, L-Tryptophan, Vitamin B6, Vitamin B1.<br>The product is gluten- and lactose-free.	1 sachet	Essential Aminoacids	Improvement of muscle health in adults

*Organic
